# COVID-19 and public health efforts in Mongolia: A lesson maybe learned?

**DOI:** 10.1017/cts.2020.510

**Published:** 2020-07-13

**Authors:** Tumenbayar Bayasgalan, Erdembileg Anuurad, Enkhmaa Byambaa

**Affiliations:** 1 Department of Endocrinology, Mongolian National University of Medical Sciences, Ulaanbaatar, Mongolia; 2 Office of Research, School of Medicine, University of California Davis, Davis, CA, USA; 3 Department of Internal Medicine, School of Medicine, University of California Davis, Davis, CA, USA

**Keywords:** Coronavirus, novel virus, new infection, spread control measurements, Asia

Geographically, Mongolia is one of the closest countries to Hubei Province, China, where the outbreak of novel coronavirus disease (COVID-19) originated. Mongolia, a landlocked country between China and Russia, has a population of over three million, of which approximately half is concentrated in the capital city Ulaanbaatar. While the average population density across the country is low (~5 per square mile), this estimation reaches to >700 per square mile in Ulaanbaatar and adjacent suburban areas. Despite these facts, at present, Mongolia reports no deaths from COVID-19 with ~200 known cases [1]. Here, we describe the factors underlying this paradoxical situation based on information exchanged between investigators at the University of California Davis and the Mongolian National University of Medical Sciences. The investigators (authors) have been collaborating to study diabetes in Mongolia within the framework of an established memorandum of understanding between the two universities. We postulate that multiple factors associated with government decisions and a series of public health measures have played crucial roles.

During the cold season, respiratory tract infections in Mongolia rise sharply in October and continue to stay high throughout March. Considering the lack of additional hospital beds and an effective treatment (vaccine) for COVID-19, the Mongolian government in consort with its public health system began implementing a series of prevention and control measures. A timeline based on resolutions and information from official sources [2,3] depicts the following picture.

The first step was taken on January 24, 2020, with the State Emergency Commission imposing a quarantine regimen [2]. Specific measures included closing of all schools (K–12, colleges, universities, etc.), switching to cyber-learning (distribution of CDs, lesson broadcasting on 17 TV channels, etc.) and instructing the authorities to be prepared for possible outbreaks. The border crossing points (BCPs) with China were closed (except for emergency/certain need-based circumstances) on February 1; designated shelters were created. The commission issued two statements: one to the Ministry of Health to ensure the readiness of medical services, to call in all resident doctors in duty, if necessary, and provide citizens with an official source of up-to-date information [3]; another to the Ministry of Education, Culture, Science and Sports regarding the closure of schools until March 2. The Public Services and Transportation Agency and Municipal Government of Ulaanbaatar were tasked with restricting the organization of public events and improving hygiene in public transportation. The government employed various sources (TV, internet, street announcements via loudspeakers, mobile phones, radio stations, etc.) to inform citizens to wear face masks, wash hands regularly, isolate themselves at home if they show signs of infection, and avoid large gatherings and unnecessary hospital visits [2].

In February, due to the increase in infections during the traditional New Year in China [4], the Mongolian government canceled the celebration of “*Tsagaan Sar*”, the Lunar NewYear (a national holiday), closed intercity roads and passenger trains, and endorsed measures to prevent infections [3]. Decisions were made to produce face masks domestically, take paid leave or work part time for parents, and close shops/restaurants for children. A special duty aircraft was tasked to repatriate Mongolians from China, South Korea, and Japan, followed by a 14-day quarantine at designated shelters.

On March 10, the first COVID-19 case was registered by a French national [3,5]. People who came into contact with the individual were identified through contact tracing methods [video surveillance (CCTV camera), interviews, questioning, etc.] by tracking all routes taken by the French national, including hotels, shops, restaurants, transportation, and workplaces; all close contacts (~120) were put under strict quarantine; extensive disinfecting measures took place immediately. As part of the disaster prevention measures, the quarantine period and school suspension were extended until June 30. Mongolia closed all BCPs including those with Russia for travelers on March 12; however, the commission [2] issued an order on March 23 to allow vulnerable groups (people with health issues or children, seniors, pregnant women, and students lacking financial support) to return home through few BCPs. In April, the quarantine period for returnees was extended to 21 days, and a community police unit to monitor street crowds was formed. As of April 30, there were 38 registered COVID-19 cases (35 Mongolians, 3 foreigners); no known local transmission or lethal cases [2,3]. In May, the number of infections in Russia rose dramatically, contributing to the increase in registered COVID-19 cases to 135 on May 16 and 179 on May 30 [3]. As of June 11, there were 194 registered COVID-19 cases with no evidence of local transmission or deaths [3].

An interesting fact that may have played a role in the COVID-19 confinement is that the Mongolian health system has a unique experience in dealing with certain high-risk infections (plague, cholera) [6]. When outbreaks of these infections occur, the health system and government workers team up to track, isolate, and treat the infected people, leading to quick successful outcomes.

This is a brief story of how a small country located between China and Russia has been dealing with COVID-19. It is likely that the current situation in Mongolia is a result of many measures undertaken by the government and public health system, facilitated by its experience with other infections and support from the community. We recognize limitations inherent to public surveillance reporting and a possibility for asymptomatic transmissions; however, there still may be a lesson that others can learn from the way Mongolia handled COVID-19 up until now.
